# Wolff-Parkinson-White Apresentado como QRS Alternante e Outros Diagnósticos Diferenciais em uma Grande Coorte de Triagem de ECG Pré-Participação

**DOI:** 10.36660/abc.20220081

**Published:** 2022-11-11

**Authors:** Daniel Y. Z. Lim, Wilbert H. H. Ho, Luokai Wang, Wee Kiat Ang, Nishanth Thiagarajan, Gerald GR Sng, Hankun Wang, Wesley TW Loo, Lim Huai Yang, Weien Chow, Terrance J Chua, Tee Joo Yeo, Paul Lim, Thuan Tee Daniel Chong

**Affiliations:** 1 Medical Classification Centre Central Manpower Base Singapore Armed Forces Cingapura Medical Classification Centre, Central Manpower Base, Singapore Armed Forces, Cingapura; 2 HQ Medical Corps Singapore Armed Forces Cingapura HQ Medical Corps, Singapore Armed Forces, Cingapura; 3 Department of Cardiology National Heart Centre Singapore Cingapura Department of Cardiology, National Heart Centre Singapore, Cingapura; 4 Department of Cardiology National University Heart Centre Singapore Cingapura Department of Cardiology, National University Heart Centre Singapore, Cingapura; 5 University Medicine Cluster National University Health System Cingapura University Medicine Cluster, National University Health System, Cingapura

**Keywords:** Síndrome de Wolf-Parkinson-White, Síndrome do Complexo PR curto-QRS normal, Pré-Excitação Tipo Mahaim, Eletrocardiografia/métodos, Eletrocardiografia/diagnóstico

## Abstract

**Fundamento:**

A síndrome de Wolff-Parkinson-White (WPW) é uma condição pró-arrítmica que pode exigir restrição de atividades extenuantes e é caracterizada por sinais de ECG, incluindo ondas delta. Observamos casos de padrões intermitentes de WPW apresentando-se como QRS alternante (‘WPW alternante’) em uma grande coorte de triagem de ECG pré-participação de homens jovens que se candidataram ao recrutamento militar.

**Objetivos:**

Nosso objetivo foi determinar o padrão de WPW alternante, as características do caso e a prevalência de outros diagnósticos diferenciais relevantes apresentando-se como alternância de QRS em um ambiente de pré-participação.

**Métodos:**

Cento e vinte e cinco mil cento e cinquenta e oito recrutas militares do sexo masculino prospectivos foram revisados de janeiro de 2016 a dezembro de 2019. Uma revisão de prontuários médicos eletrônicos identificou casos de WPW alternante e padrões ou síndrome de WPW. A revisão de prontuários médicos eletrônicos identificou casos de diagnósticos diferenciais relevantes que podem causar alternância de QRS.

**Resultados:**

Quatro indivíduos (2,2%) apresentaram WPW alternante em 184 indivíduos com diagnóstico final de padrão ou síndrome de WPW. Dois desses indivíduos manifestaram sintomas ou achados eletrocardiográficos compatíveis com taquicardia supraventricular. A prevalência geral de WPW alternante foi de 0,003%, e a prevalência de WPW foi de 0,147%. As WPW alternantes representaram 8,7% dos indivíduos com QRS alternantes, e QRS alternantes tiveram prevalência de 0,037% em toda a população.

**Conclusões:**

A WPW alternante é uma variante da WPW intermitente, que compreendeu 2,2% dos casos de WPW em nossa coorte de triagem pré-participação. Não indica necessariamente um baixo risco de taquicardia supraventricular. Deve ser reconhecido na triagem de ECG e distinguido de outras patologias que também apresentam QRS alternantes.

## Introdução

A avaliação física pré-participação antes de atividades extenuantes, como esportes, pode permitir a detecção de condições médicas potencialmente desqualificantes, como anormalidades cardíacas graves. A triagem eletrocardiográfica (ECG) é uma das modalidades utilizadas nessas avaliações para detectar condições pró-arrítmicas. É recomendado por várias sociedades profissionais e diretrizes,^1 - 3^ com critérios de interpretação, incluindo os critérios^4^ da European Society of Cardiology (ESC) 2010, Seattle Criteria,^5^ Refined Criteria,^6^ e, mais recentemente, os Critérios Internacionais para interpretação de ECG em Atletas.^7^

Wolff-Parkinson-White (WPW) é uma condição potencialmente pró-arrítmica que pode ser detectada na triagem de ECG. A anormalidade subjacente é uma via acessória que permite a condução dos átrios para os ventrículos, contornando o nó atrioventricular. Essa pré-excitação se manifesta como ondas delta, intervalo PR curto, intervalo QRS prolongado e anormalidades de repolarização no ECG. O padrão WPW no ECG em combinação com taquiarritmia sintomática constitui a síndrome WPW. Indivíduos com WPW requerem revisão e estratificação de risco antes de participar de atividades extenuantes. O padrão WPW convencional é prontamente reconhecível pela pré-excitação em cada batimento, mas raramente pode apresentar pré-excitação intermitente em batimentos alternados (ou seja, com QRS alternante, definido pela alternância de amplitude, morfologia ou duração do complexo QRS). Esse fenômeno pode dificultar o reconhecimento, existindo apenas relatos de casos isolados do fenômeno ‘WPW alternante’ na literatura.^8 - 10^ Esses casos foram identificados esporadicamente, em vez de coletados sistematicamente.

A alternância WPW também deve ser distinguida de outras etiologias de alternância QRS. Em situações de emergência, um diagnóstico diferencial chave que é classicamente descrito é o tamponamento pericárdico (onde há QRS alternante devido à oscilação mecânica do ápice cardíaco em vez da patologia das vias de condução intrínsecas). Outras situações em que o QRS alternante pode ocorrer em ritmos não estimulados incluem pausa sinusal intermitente/bloqueio de saída com escape ventricular ou escape juncional com bloqueio de ramo (BR), BR intermitente em batimentos alternados, bigeminismo atrial com condução aberrante e bigeminismo ventricular. Também pode ocorrer durante taquiarritmias, como taquicardia ventricular bidirecional e taquicardia supraventricular em altas frequências (por exemplo, taquicardia por reentrada atrioventricular).

Nenhuma literatura examinou sistematicamente a prevalência de WPW alternante ou QRS alternante em um ambiente de pré-participação, não emergencial. No entanto, continua sendo necessário que os médicos que realizam a triagem de ECG reconheçam corretamente WPW alternante como uma forma de WPW, distingam-no de outras causas de QRS alternante e, a partir daí, determinem se a causa subjacente justifica a exclusão da participação.

As Forças Armadas de Cingapura realizaram triagem universal de ECG pré-participação antes do alistamento militar para todos os jovens cingapurianos do sexo masculino para determinar a aptidão cardíaca.^11 - 13^ Nosso objetivo principal foi determinar sistematicamente a prevalência do padrão WPW alternante e as características relevantes do caso neste cenário de pré-participação, não emergencial. Nosso objetivo secundário foi determinar sistematicamente a prevalência de outros diagnósticos diferenciais relevantes apresentando-se como QRS alternante no mesmo cenário.

## Métodos

Cento e vinte e cinco mil cento e cinquenta e oito recrutas militares do sexo masculino em potencial foram revisados de janeiro de 2016 a dezembro de 2019 como parte de sua determinação de aptidão para se alistar no serviço militar. Todos os indivíduos compareceram ao mesmo serviço centralizado e realizaram ECG de 12 derivações em decúbito dorsal. Os ECGs foram informados por médicos treinados usando um algoritmo padronizado baseado nos Critérios Internacionais.^7^

Durante este período, observamos quatro casos de WPW alternante no ECG apresentado. Revisamos as referências eletrônicas ao centro terciário nacional de cardiologia para suspeita de padrões de ECG de WPW e os registros médicos eletrônicos dos 184 indivíduos que receberam um novo padrão de WPW ou diagnóstico de síndrome. Não foram identificados outros casos de alternância WPW. Separadamente, identificamos 34 indivíduos que haviam sido diagnosticados com WPW antes de participar da triagem pré-participação. Esses casos foram excluídos de nossa análise porque seus ECGs não estavam disponíveis. Esses 34 indivíduos tiveram ECGs repetidos em nosso centro de triagem, dos quais nenhum apresentou WPW alternante.

Concomitantemente, identificamos indivíduos com QRS alternante de outras etiologias. Determinamos isso por dois meios: primeiro, por uma revisão da documentação de texto feita para a interpretação clínica do ECG; segundo, por uma revisão dos códigos diagnósticos para arritmias cardíacas e tamponamento pericárdico. Com o primeiro método, empregamos uma pesquisa por palavra-chave e uma revisão manual do gráfico de toda a documentação. Os termos de pesquisa para as várias condições estão listados no Apêndice 1 e são baseados no vocabulário padronizado de interpretação de ECG de nossas instalações. Extraímos os prontuários eletrônicos de indivíduos com os códigos de diagnóstico relevantes com o segundo método. Isso garantiu a identificação exaustiva de indivíduos com morfologia QRS alternante. Todos os indivíduos identificados com WPW alternantes e QRS alternantes tiveram seus ECG revisados manualmente.^14^

A aprovação para coleta e uso de dados foi concedida pelo Comitê Médico Conjunto das Forças Armadas de Cingapura e a aprovação ética foi obtida do conselho de revisão institucional local.

### Análise estatística

Adotou-se uma abordagem descritiva, calculando a prevalência de WPW alternante na população geral e indivíduos com WPW. Também calculamos a prevalência de QRS alternante na população geral e os diagnósticos individuais causadores de QRS alternante. A análise quantitativa foi feita usando Excel (Microsoft 365 Apps). Os detalhes de casos de indivíduos com WPW alternante são apresentados qualitativamente.

## Resultados

Quatro indivíduos (2,2%) apresentaram WPW alternante em 184 indivíduos com diagnóstico final de padrão ou síndrome de WPW. Em toda a coorte de triagem, a prevalência de WPW alternante foi de 0,003% e a prevalência de WPW foi de 0,147%.

**Figura 1:**
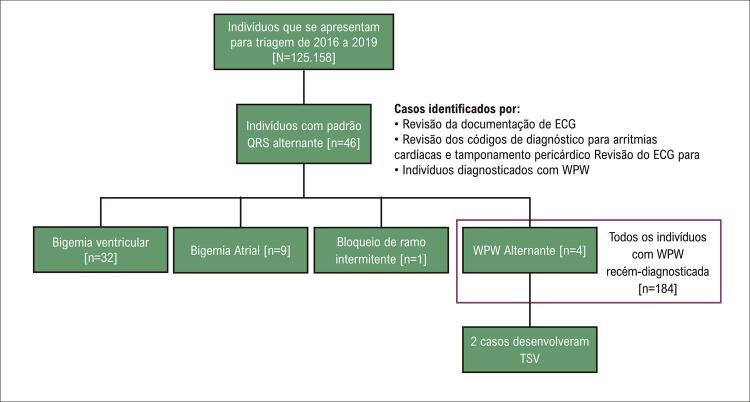
Fluxograma dos indivíduos incluídos no estudo. ECG: eletrocardiográfica; TSV: taquicardia supraventricular; WPW: Wolff-Parkinson-White..

Também identificamos outros 42 indivíduos com morfologia de QRS alternante a partir de revisão de prontuários na triagem de ECG. Este incluiu 1 indivíduo com BR intermitente em batimentos alternados, 9 indivíduos com bigeminismo atrial e 32 com bigeminismo ventricular. Nenhum indivíduo apresentou bloqueio/pausa sinusal intermitente com escape ventricular, escape juncional com BR ou taquicardia ventricular ao apresentar ECG. Nenhum indivíduo em nossa coorte teve diagnóstico ativo de tamponamento pericárdico no momento da triagem. No geral, os WPW alternantes representaram 8,7% dos indivíduos que apresentavam QRS alternantes, e os QRS alternantes tiveram uma prevalência de 0,037% em toda a população de triagem de ECG. Todos os indivíduos identificados não tinham doença cardíaca pré-existente. Essas descobertas são resumidas por meio de um diagrama de fluxo na Figura 1 .

Apresentamos na Tabela 1 os detalhes do caso dos quatro indivíduos com WPW alternantes. No momento da redação deste artigo, nenhum dos indivíduos havia consentido em estudos eletrofisiológicos invasivos e nenhum havia sofrido morte súbita cardíaca. Todos foram aconselhados a não se envolverem em atividades fisicamente extenuantes.

**Tabela 1:** Detalhes do caso WPW alternante

	Caso 1	Caso 2	Caso 3	Caso 4
ECG na triagem	Por favor, consulte o Apêndice 2 para ECGs de cada caso			
Idade	19	19	18	18
Sintomas	Palpitações Sem síncope	Palpitações Sem síncope	Palpitações Sem síncope	Sem sintomas
Presença de taquicardia supraventricular	Sim Teve palpitações consideradas clinicamente compatíveis com taquicardia supraventricular	Sim Desenvolveu taquicardia RP longo durante a consulta – diagnosticada como taquicardia atrial	Não	Não
Teste de esforço em esteira	Pré-excitado com pré-excitação intermitente	Pré-excitado com perda de pré-excitação na FC máxima de 194 bpm	Pré-excitado sem perda súbita de pré-excitação	Normal
Ecocardiograma 2D	Normal	Normal	Normal	Não realizada
Outras investigações	Holter 24 horas: pré-excitação intermitente	Nada	Holter 24 horas: pré-excitação intermitente	Nada

*ECG: eletrocardiográfica; FC: frequência cardíaca. RP: intervalo de onda R para P.*

## Discussão

A triagem de ECG pré-participação eficaz requer o reconhecimento de padrões anormais, incluindo apresentações incomuns de anormalidades de ECG. Nossa casuística de quatro indivíduos com WPW alternantes mostrou prevalência de 2,2% entre os casos de WPW e 0,003% em toda a coorte. Até onde sabemos, esta é a maior série de casos de WPW alternantes na literatura e o único estudo que determinou sistematicamente sua prevalência. Isso sugere que WPW alternante pode não ser tão raro quanto sua escassez na literatura existente sugere.

A WPW alternante é uma forma de pré-excitação intermitente, convencionalmente pensada para conferir menor risco de taquicardia supraventricular.^15^ Não observamos isso em nossa série de casos - na verdade, o caso 2 desenvolveu episódios de taquicardia supraventricular (TSV) durante a consulta de triagem. Foi cardiovertido com manobras vagais e encaminhado de emergência ao centro terciário nacional de cardiologia. O caso 4 apresentou palpitações recorrentes que o cardiologista responsável considerou compatíveis com TSV paroxística. Evidência de Escudero et al.,^15^ em um recente estudo de WPW multicêntrico pediátrico^16^ sugere que a pré-excitação intermitente não é totalmente isenta de risco, e esses indivíduos ainda podem ter vias acessórias subjacentes com alto risco de desenvolver TSV. Portanto, é importante que o padrão WPW alternante seja adequadamente reconhecido no momento da triagem de ECG e não seja erroneamente considerado benigno. Deve ser concedido o mesmo tratamento que qualquer outro caso de WPW.

O reconhecimento de WPW alternante pode ser confundido por outras patologias que apresentam o padrão QRS alternante. Como esperado de uma coorte jovem, pré-participação, não emergencial, não foram diagnosticados casos de patologias agudas graves, como taquicardia ventricular bidirecional ou tamponamento cardíaco. O bigeminismo ventricular foi a patologia alternativa mais comum, seguido pelo bigeminismo atrial e BR intermitente em batimentos alternados. Exemplos de ECGs de tais condições coletadas de indivíduos nesta arte de coorte incluídos no Apêndice 3. A única maneira de distinguir WPW dessas outras patologias é examinar cuidadosamente o ECG para ondas delta. Como tal, os médicos examinadores devem estar atentos a esses outros diagnósticos diferenciais e considerar ECGs seriados para elicitação adicional de ondas delta.

A principal força do nosso estudo é que foi uma pesquisa abrangente, em toda a população, realizada ao longo de vários anos. É o primeiro estudo a examinar sistematicamente a epidemiologia do padrão WPW alternante no cenário de triagem de ECG e a documentar a epidemiologia de importantes diagnósticos diferenciais que também podem se apresentar com QRS alternante. Isso ajudará os médicos que realizam a triagem de ECG a chegar a um diagnóstico diferencial relevante para o sinal visualmente marcante de QRS alternante, pois as patologias observadas não são as mesmas descritas em configurações de emergência.

Nosso estudo tem algumas limitações importantes. Primeiro, os ECGs do estudo foram revisados por diferentes médicos, e a equipe do estudo não revisou pessoalmente todos os ECGs do banco de dados. Pode haver variação interindividual na interpretação do ECG entre os médicos. Em segundo lugar, notamos que a WPW não tem predileção por gênero em grupos etários pediátricos,^17^ mas é conhecida por ter uma predominância masculina em adultos.^18^ A prevalência relativa de nossa coorte de adolescentes do sexo masculino pode, portanto, não ser totalmente generalizável para indivíduos do sexo feminino. No entanto, o reconhecimento correto do sinal de ECG alternante e WPW alternante ainda seria importante para indivíduos do sexo feminino submetidos à triagem de ECG. Terceiro, não podemos confirmar nenhum mecanismo eletrofisiológico específico de WPW alternante, pois nenhum dos indivíduos identificados consentiu em um estudo eletrofisiológico invasivo. Finalmente, não podemos comentar sobre o risco de morte súbita cardíaca em longo prazo para os indivíduos com WPW ou WPW alternantes neste estudo, uma vez que os indivíduos examinados tinham sido diagnosticados apenas nos últimos anos. Estudos futuros podem incluir um acompanhamento prolongado desses indivíduos para melhor verificar seu risco de morte súbita cardíaca.

## Conclusão

WPW alternante é uma apresentação variante de WPW intermitente, que ocorreu em 2,2% dos casos de WPW em uma coorte de triagem pré-participação. Não indica necessariamente um baixo risco de taquicardia supraventricular. Portanto, deve ser reconhecida na triagem de ECG e distinguido de outras patologias que também apresentam QRS alternante. Nos diagnósticos diferenciais comuns de QRS alternante observados em nossa pré-participação, a coorte não emergencial incluiu bigeminismo ventricular, bigeminismo atrial e BR intermitente em batimentos alternados.

## *Material suplementar

Para informação adicional, por favor, clique aqui


