# Supplementary material for: Wolff-Parkinson-White Apresentado como QRS Alternante e Outros Diagnósticos Diferenciais em uma Grande Coorte de Triagem de ECG Pré-Participação
Source: Arq Bras Cardiol. 2022 Nov 11;119(6):940–5. [Article in Portuguese] doi: 10.36660/abc.20220081 (PMC9814817; doi:10.36660/abc.20220081)
Supplement: Supplementary file 1 [file 2022-0081-supp-material.pdf]

Appendix 1: Keyword search terms

‘alternating’, ‘alternans’, ‘alternate’
‘bigeminy’, ‘atrial bigeminy’, ‘ventricular bigeminy’
‘bundle branch block’, ‘BBB’
‘ventricular tachycardia’, ‘VT’
‘supraventricular tachycardia’, ‘SVT

Appendix 2: WPW Alternans ECGs

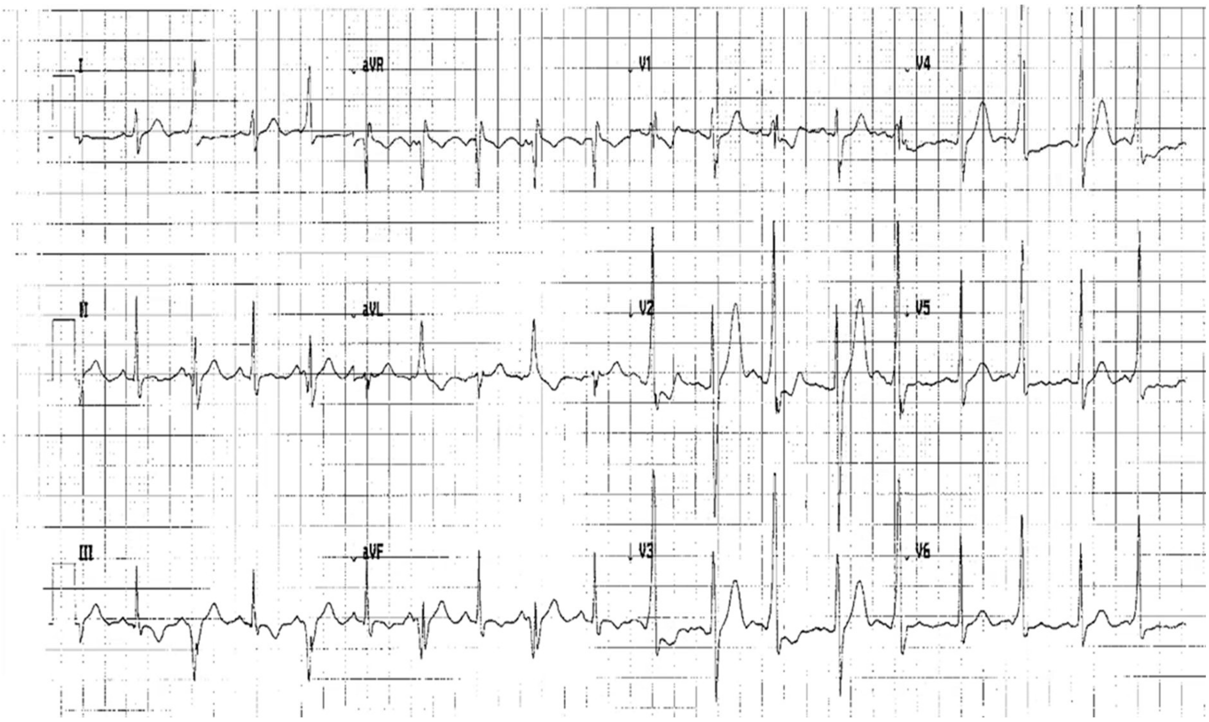

Appendix 2.1: Case 1

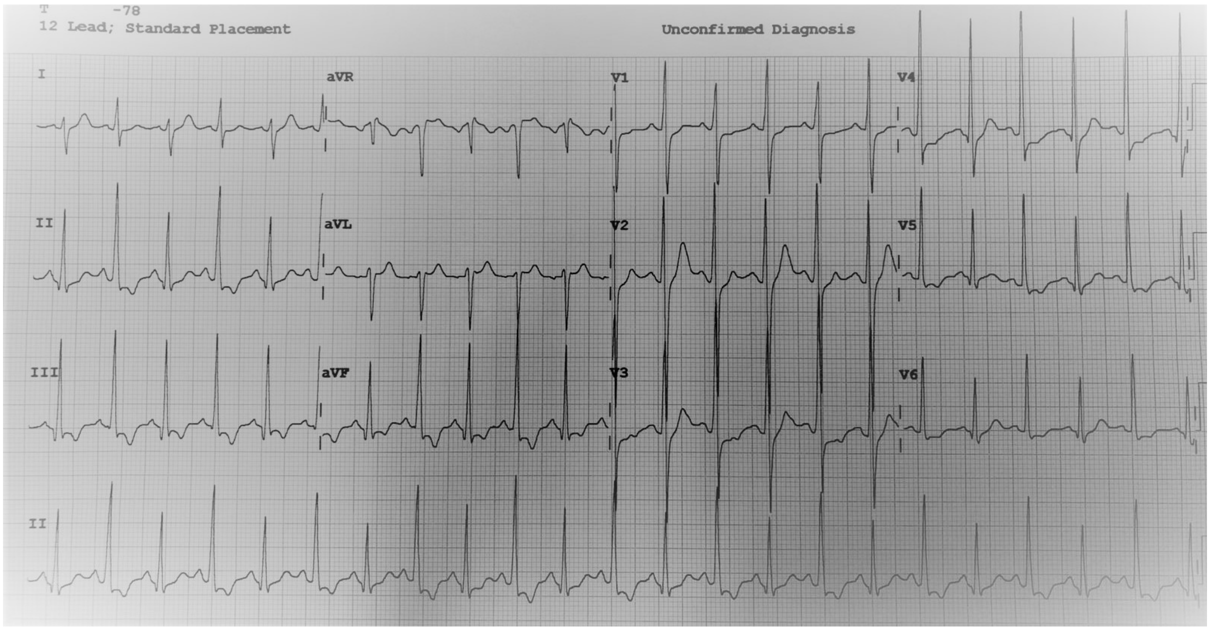

Appendix 2.2: Case 2

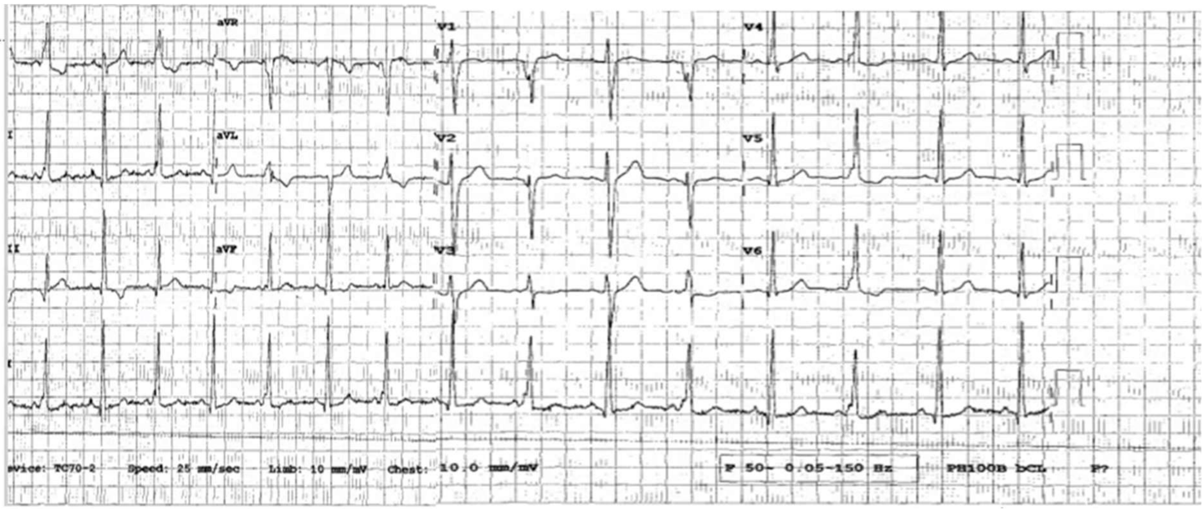

Appendix 2.3: Case 3

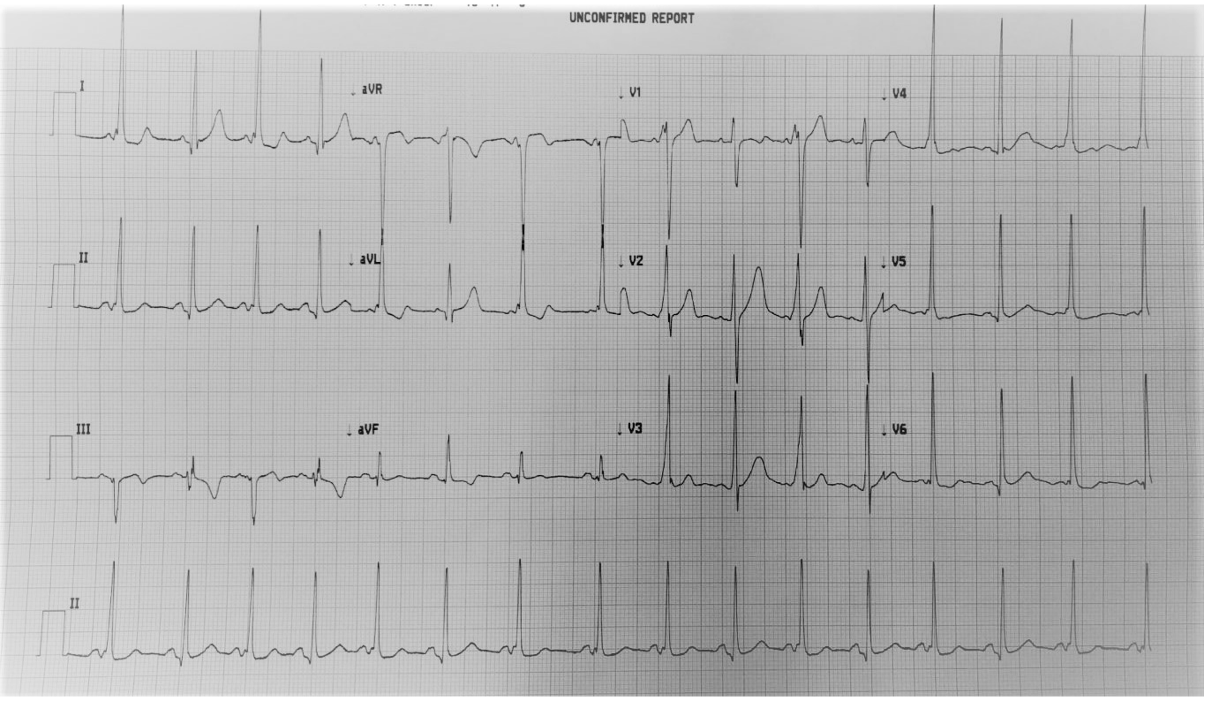

Appendix 2.4: Case 4

Appendix 3: Example ECGs of Other Differential Diagnoses of QRS Alternans

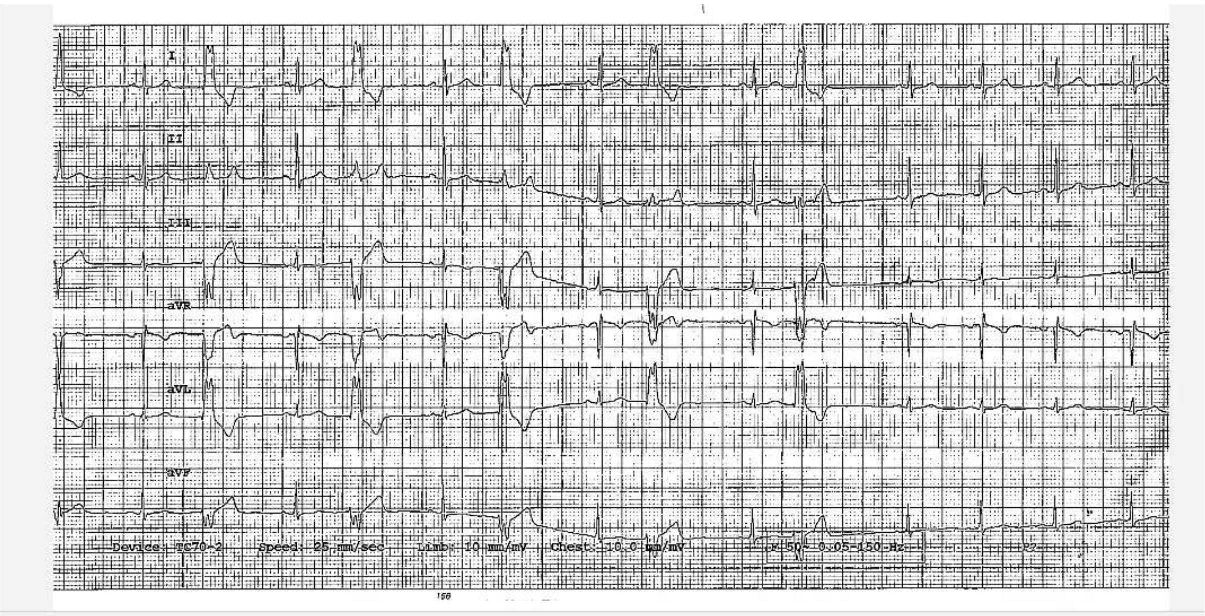

Appendix 3.1: Ventricular bigeminy

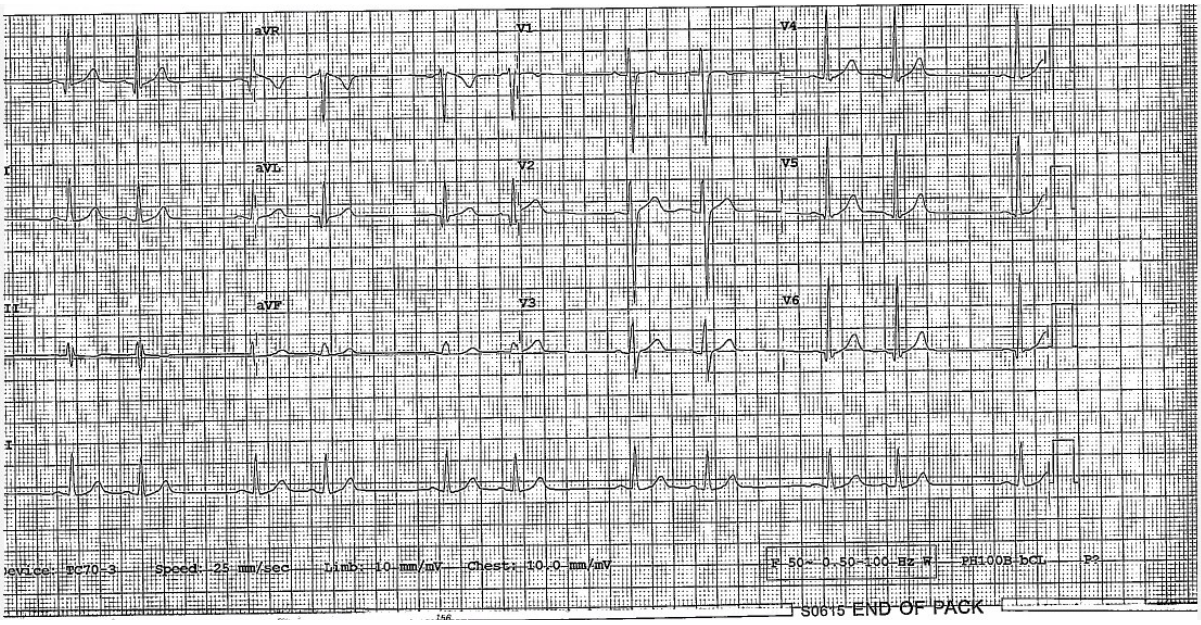

Appendix 3.2: Atrial bigeminy

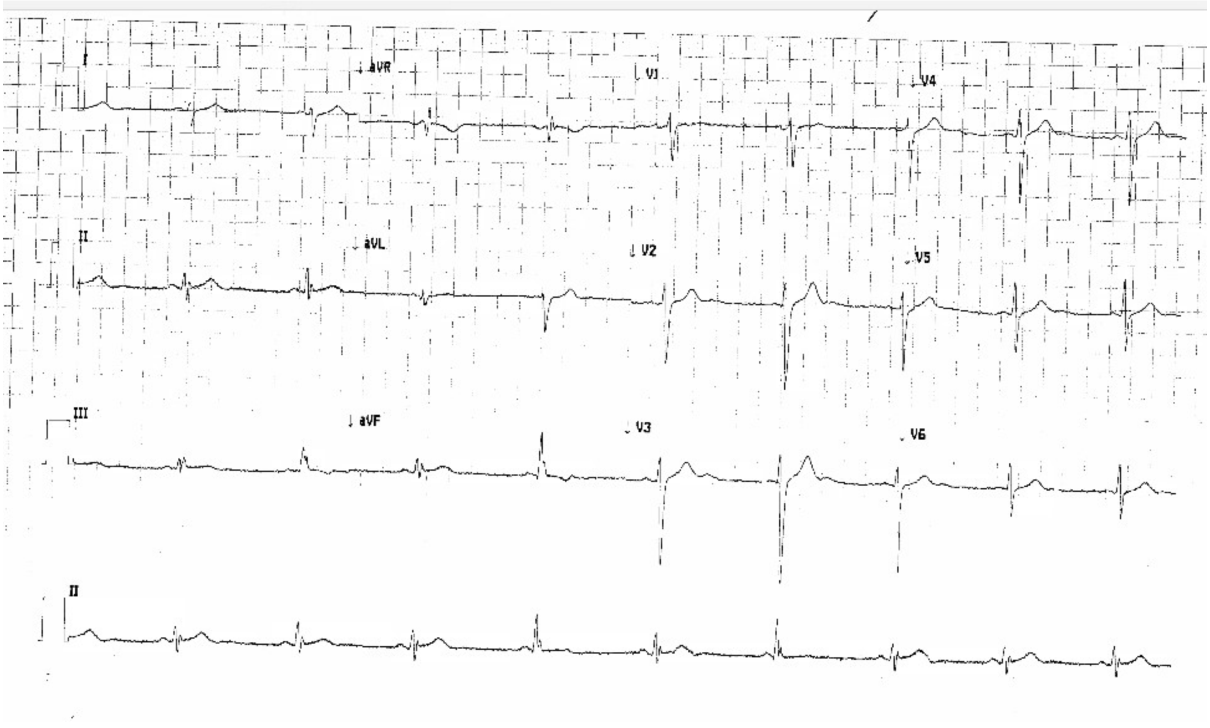

Appendix 3.3: Intermittent bundle branch block on alternate beats
