# Supplementary material for: Wolff-Parkinson-White Apresentado como QRS Alternante e Outros Diagnósticos Diferenciais em uma Grande Coorte de Triagem de ECG Pré-Participação
Source: Arq Bras Cardiol. 2022 Nov 11;119(6):940–5. [Article in Portuguese] doi: 10.36660/abc.20220081 (PMC9814817; doi:10.36660/abc.20220081)
Supplement: Translation

# Translation: Wolff-Parkinson-White Presenting as QRS Alternans and Other Differential Diagnoses in a Large Pre-Participation ECG Screening Cohort

**Authors:** Daniel Y. Z. Lim, Wilbert H. H. Ho, Luokai Wang, Wee Kiat Ang, Nishanth Thiagarajan, Gerald GR Sng, Hankun Wang, Wesley TW Loo, Lim Huai Yang, Weien Chow, Terrance J Chua, Tee Joo Yeo, Paul Lim, Thuan Tee Daniel Chong

**Affiliations:** 1 Singapore Medical Classification Centre, Central Manpower Base, Singapore Armed Forces, Singapore; 2 Singapore HQ Medical Corps, Singapore Armed Forces, Singapore; 3 Singapore Department of Cardiology, National Heart Centre Singapore, Singapore; 4 Singapore Department of Cardiology, National University Heart Centre Singapore, Singapore; 5 Singapore University Medicine Cluster, National University Health System, Singapore

**Keywords:** Wolff-Parkinson-White Syndrome, Lown – Ganong – Levine Syndrome, Pre-Excitation, Mahaim-Type, Electrocardiography/methods, Electrocardiography/diagnosis

## Abstract

**Background:**

Wolff-Parkinson-White (WPW) syndrome is a proarrhythmic condition that may require restriction from strenuous activities and is characterized by ECG signs, including delta waves. We observed cases of intermittent WPW patterns presenting as QRS alternans (‘WPW alternans’) in a large pre-participation ECG screening cohort of young men reporting for military conscription.

**Objectives:**

We aimed to determine the WPW alternans pattern, case characteristics, and the prevalence of other relevant differential diagnoses presenting as QRS alternans in a pre-participation setting.

**Methods:**

One hundred twenty-five thousand one hundred fifty-eight prospective male military recruits were reviewed from January 2016 to December 2019. A review of electronic medical records identified cases of WPW alternans and WPW patterns or syndrome. Reviewing electronic medical records identified cases of relevant differential diagnoses that might cause QRS alternans.

**Results:**

Four individuals (2.2%) had WPW alternans out of 184 individuals with a final diagnosis of WPW pattern or syndrome. Two of these individuals manifested symptoms or ECG findings consistent with supraventricular tachycardia. The overall prevalence of WPW alternans was 0.003%, and the prevalence of WPW was 0.147%. WPW alternans represented 8.7% of individuals presenting with QRS alternans, and QRS alternans had a prevalence of 0.037% in the entire population.

**Conclusions:**

WPW alternans is a variant of intermittent WPW, which comprised 2.2% of WPW cases in our pre-participation screening cohort. It does not necessarily indicate a low risk for supraventricular tachycardia. It must be recognized at ECG screening and distinguished from other pathologies that also present with QRS alternans.

## Introduction

Preparticipation physical evaluation prior to strenuous activities such as sports may allow for the detection of potentially disqualifying medical conditions, such as serious cardiac abnormalities. Electrocardiographic (ECG) screening is one of the modalities used in such evaluations to detect proarrhythmic conditions. It is recommended by multiple professional societies and guidelines,^1 - 3^ with interpretation criteria including the European Society of Cardiology (ESC) 2010 criteria,^4^ Seattle Criteria,^5^ Refined Criteria (2014)^6^ and most recently, the International Criteria for ECG interpretation in Athletes (2017).^7^

Wolff-Parkinson-White (WPW) is a potentially proarrhythmic condition that can be detected on ECG screening. The underlying abnormality is an accessory pathway that allows conduction from the atria to the ventricles, bypassing the atrioventricular node. This pre-excitation manifests as delta waves, short PR interval, prolonged QRS interval, and repolarization abnormalities on ECG. The WPW pattern on ECG in combination with symptomatic tachyarrhythmia constitutes WPW syndrome. Individuals with WPW require review and risk stratification before participating in strenuous activities. The conventional WPW pattern is readily recognizable by pre-excitation on every beat but can rarely present intermittent pre-excitation on alternate beats (i.e., with QRS alternans, defined by alternating amplitude, morphology, or duration of the QRS complex). This phenomenon may make recognition challenging, and only isolated case reports exist of the ‘WPW alternans’ phenomenon in the literature.^8 - 10^ These cases were sporadically identified rather than systematically collected.

WPW alternans must also be distinguished from other etiologies of QRS alternans. In emergency settings, a key differential diagnosis that is classically described is pericardial tamponade (where there are QRS alternans due to mechanical swinging of the cardiac apex rather than the pathology of the intrinsic conduction pathways). Other situations in which QRS alternans may occur in non-paced rhythms include intermittent sinus pause/ exit block with ventricular escape or junctional escape with bundle branch block (BBB), intermittent BBB on alternate beats, atrial bigeminy with aberrant conduction and ventricular bigeminy. It may also occur during tachyarrhythmias such as bidirectional ventricular tachycardia and supraventricular tachycardias at high rates (e.g., atrioventricular re-entrant tachycardia).

No literature has systematically examined the prevalence of WPW alternans or QRS alternans in a pre-participation, non-emergency setting. However, it remains necessary for medical providers conducting ECG screening to correctly recognize WPW alternans as a form of WPW, distinguish it from other causes of QRS alternans, and thence determine if the underlying cause warrants exclusion from participation.

The Singapore Armed Forces have conducted universal pre-participation ECG screening before military enlistment for all young male Singaporeans to determine cardiac fitness.^11 - 13^ Our primary aim was to systematically determine the prevalence of the WPW alternans pattern and the relevant case characteristics in this pre-participation, non-emergency setting. Our secondary aim was to systematically determine the prevalence of other relevant differential diagnoses presenting as QRS alternans in the same setting.

## Methods

One hundred twenty-five thousand one hundred fifty-eight prospective male military recruits were reviewed from January 2016 to December 2019 as part of their determination of fitness to enlist in military service. All individuals attended the same centralized facility and had a supine resting 12-lead ECG. The ECGs were reported by trained clinicians using a standardized algorithm based on International Criteria.^7^

During this period, we observed four cases of WPW alternans on the presenting ECG. We reviewed electronic referrals to the national cardiology tertiary center for suspected WPW ECG patterns and the electronic medical records of the 184 individuals who had received a new WPW pattern or syndrome diagnosis. No other cases of WPW alternans were identified. Separately, we identified 34 individuals who had been diagnosed with WPW prior to attending the pre-participation screening. These cases were excluded from our analysis because their presenting ECGs were unavailable. These 34 individuals had repeat ECGs at our screening center, of which none displayed WPW alternans.

Concurrently, we identified individuals presenting with QRS alternans arising from other etiologies. We determined this through two means: First, by a review of text documentation made for clinical ECG interpretation; Second, by a review of diagnostic codes for cardiac arrhythmias and pericardial tamponade. With the first method, we employed a keyword search and a manual chart review of all documentation. The search terms for the various conditions are listed in Appendix 1 and are based on our facility’s standardized ECG interpretation vocabulary. We extracted the electronic medical records for individuals with the relevant diagnosis codes with the second method. This ensured exhaustive identification of individuals with QRS alternans morphology. All individuals identified with WPW alternans and QRS alternans had their ECG manually reviewed.

Approval for data collection and use was granted by the Singapore Armed Forces Joint Medical Committee, and ethical approval was obtained from the local institutional review board.

### Statistical analysis

We adopted a descriptive approach, calculating the prevalence of WPW alternans in the overall population and individuals with WPW. We also calculated the prevalence of QRS alternans in the overall population and the individual diagnoses causing QRS alternans. Quantitative analysis was done using Excel (Microsoft 365 Apps). Case details of individuals with WPW alternans are qualitatively presented.

## Results

Four individuals (2.2%) had WPW alternans out of 184 individuals with a final diagnosis of WPW pattern or syndrome. In the entire screening cohort, the prevalence of WPW alternans was 0.003%, and the prevalence of WPW was 0.147%.

We also identified 42 other individuals with QRS alternans morphology from medical records review on screening ECG. This comprised 1 individual with intermittent BBB on alternating beats, 9 individuals with atrial bigeminy, and 32 with ventricular bigeminy. No individuals had intermittent sinus pause/exit block with ventricular escape, junctional escape with BBB, or ventricular tachycardia on presenting ECG. No individuals in our cohort had an active diagnosis of pericardial tamponade at the point of screening. Overall, WPW alternans represented 8.7% of individuals presenting with QRS alternans, and QRS alternans had a prevalence of 0.037% in the entire ECG screening population. All of the individuals identified did not have pre-existing cardiac disease. These findings are summarized via a flow diagram in Figure 1 .

**Figure 1:** Flow diagram of individuals included in the study. ECG: electrocardiographic; WPW: Wolff-Parkinson-White; SVT: supraventricular tachycardia.

We present in Table 1 the case details of the four individuals with WPW alternans. At the time of writing, none of the individuals had consented to invasive electrophysiology studies, and none had experienced any sudden cardiac death. All were advised not to engage in physically strenuous activities.

**Table 1:** WPW alternans case details

	Case 1	Case 2	Case 3	Case 4
ECG at screening	Please refer to Appendix 2 for ECGs of each case			
Age	19	19	18	18
Symptoms	Palpitations No syncope	Palpitations No syncope	Palpitations No syncope	No symptoms
Presence of supraventricular tachycardia	Yes Had palpitations deemed clinically compatible with supraventricular tachycardia	Yes Developed long RP tachycardia during consult – diagnosed as atrial tachycardia	No	No
Treadmill exercise test	Pre-excited with intermittent pre-excitation	Pre-excited with loss of pre-excitation at maximal HR of 194 bpm	Pre-excited with no sudden loss of pre-excitation	Normal
2D Echocardiogram	Normal	Normal	Normal	Not performed
Other investigations	24 hour Holter: intermittent pre-excitation	Nil	24 hour Holter: intermittent pre-excitation	Nil

*ECG: Electrocardiographic ; HR: heart rate; RP: R to P wave interval.*

## Discussion

Effective pre-participation ECG screening requires recognizing abnormal patterns, including uncommon presentations of ECG abnormalities. Our case series of four individuals with WPW alternans showed a prevalence of 2.2% among WPW cases and 0.003% in the entire cohort. To our knowledge, this is the largest case series of WPW alternans in the literature and the only study that has systematically determined its prevalence. It suggests that WPW alternans may not be as rare as its paucity in the extant literature suggests.

WPW alternans is a form of intermittent pre-excitation, which is conventionally thought to confer a lower risk of supraventricular tachycardia.^15^ We did not observe this in our case series - in fact, Case 2 developed episodes of supraventricular tachycardia (SVT) during the screening consultation. He was cardioverted with vagal maneuvers and emergently referred to the national cardiology tertiary center. Case 4 had recurrent palpitations that the attending cardiologist deemed consistent with paroxysmal SVT. Evidence from Escudero et al.^15^ in a recent multicenter pediatric WPW study^16^ suggests that intermittent pre-excitation is not entirely risk-free, and such individuals may still have underlying accessory pathways at high risk for developing the SVT. It is thus important that the WPW alternans pattern is adequately recognized at the point of ECG screening and not mistakenly deemed benign. It should be accorded the same treatment as any other case of WPW.

Recognition of WPW alternans may be confounded by other pathologies presenting with the QRS alternans pattern. As expected of a young, pre-participation, non-emergency cohort, no cases of severe acute pathologies such as bidirectional ventricular tachycardia or cardiac tamponade were diagnosed. Ventricular bigeminy was the most common alternative pathology, followed by atrial bigeminy and intermittent BBB on alternate beats. Example ECGs of such conditions gathered from individuals in this cohort art included in Appendix 3. The only way to distinguish WPW from these other pathologies is to examine the ECG for delta waves carefully. As such, examiner physicians must be alert to these other differential diagnoses and consider serial ECGs for further elicitation of delta waves.

The main strength of our study is that it was a comprehensive, population-wide survey conducted over multiple years. It is the first study to systematically examine the epidemiology of the WPW alternans pattern in the ECG screening setting and to document the epidemiology of important differential diagnoses that can also present with QRS alternans. This will aid clinicians performing ECG screening in arriving at a relevant differential diagnosis for the visually striking sign of QRS alternans, as the pathologies observed are not the same as those described in emergency settings.

Our study has some important limitations. First, the ECGs in the study were reviewed by different clinicians, and the study team did not personally review every ECG in the database. There may be inter-individual variation in ECG interpretation between clinicians. Second, we note that WPW has no gender predilection in pediatric age groups^17^ but is known to have a male predominance in adults.^18^ The relative prevalence of our cohort of adolescent males may thus not be entirely generalizable to female individuals. However, correct recognition of the alternans ECG sign and WPW alternans would still be important for female individuals undergoing ECG screening. Third, we cannot confirm any specific electrophysiologic mechanism of WPW alternans, as none of the individuals identified consented to an invasive electrophysiologic study. Finally, we cannot comment on the long-term sudden cardiac death risk for the individuals with WPW or WPW alternans in this study, given that the individuals examined had only been diagnosed within the last few years. Future studies may include extended follow-up for such individuals to ascertain their sudden cardiac death risk better.

## Conclusion

WPW alternans is a variant presentation of intermittent WPW, which occurred in 2.2% of WPW cases in a pre-participation screening cohort. It does not necessarily indicate a low risk for supraventricular tachycardia. Hence, it must be recognized at ECG screening and distinguished from other pathologies that also present with QRS alternans. Common differential diagnoses of QRS alternans observed in our pre-participation, the non-emergency cohort included ventricular bigeminy, atrial bigeminy and intermittent BBB on alternate beats.

## * Supplemental Materials

For additional information, please, click here
